# Effect of age and sex differences on the abundances of neuronal, glial, and endothelial cells in non-diseased brain tissue

**DOI:** 10.1371/journal.pone.0313855

**Published:** 2024-12-19

**Authors:** Arttu Autio-Kimura, Tapio Nevalainen, Mikko Hurme

**Affiliations:** 1 Faculty of Medicine and Health Technology, Tampere University, Tampere, Finland; 2 Gerontology Research Center (GEREC), Tampere, Finland; 3 Tampere University Hospital, Tampere, Finland; State University of New York Upstate Medical University, UNITED STATES OF AMERICA

## Abstract

Neuroinflammatory and neurodegenerative diseases are influenced by the complex interplay of different cell types within the brain, and understanding the proportions and dynamics of neuronal, glial, and endothelial cells is crucial for deciphering the mechanisms of these diseases. Certain risk factors, such as age and sex differences, are thought to play a significant role in the susceptibility, progression, and response to neurological disease. Therefore, investigation of age- and sex-related differences in cell type proportions is needed to elucidate the biological basis of these diseases. Advances in sequencing technology have enabled large-scale transcriptomic studies, such as the Genotype-Tissue Expression (GTEx) project, providing valuable resources for investigating the cellular landscape of the human brain. In this analysis, we used brain sample data from the GTEx project, comprising 1646 samples with an age range of 20–70 years. The relative abundance of excitatory and inhibitory neurons, astrocytes, oligodendrocytes, microglia, and endothelial cells was estimated from the RNA sequencing data using a deconvolution-based analysis. Spearman correlation analysis between the individuals’ calendar ages and cell type proportions revealed a statistically significant decrease in the proportion of neurons with increasing age. In contrast, the proportions of astrocytes and endothelial cells showed a significant increase. Furthermore, endothelial cells exhibited the strongest correlation coefficient, positively associating with age. In addition, the findings indicate sex-based differences in age-related changes to cell type proportions. An age-associated decrease in neuronal proportions was only observed in male donors, while no significant change was found in females. Additionally, an age-associated increase in astrocyte proportions was exclusively seen in males, whereas only females exhibited a significant increase in microglia proportions. Furthermore, we identified sex-based differences in baseline cell type proportions. Male originating samples exhibited higher proportions of excitatory neurons, while female samples showed higher proportions of microglia and endothelial cells. Our results show that both age and sex affect the proportions of cell types in non-diseased brain tissue samples. These findings contribute to our understanding of the effects of age and sex differences on the cellular composition of the brain and shed light on the potential roles of age and sex in neurological diseases.

## Introduction

Aging is a significant risk factor for many diseases. However, the underlying mechanisms are still not well understood. It has been suggested that mechanisms influencing or triggering inflammation play a role. This phenomenon is commonly referred to as inflammaging [1]. Furthermore, the link between age and neurodegenerative disease is clear, as for example in Alzheimer’s disease (AD) the most significant risk factor is advanced age [2]. Despite the overall functional decline in aging, not all age-associated changes are necessarily harmful, as some may be physiological attempts to compensate and mitigate age-associated decline. A crucial question in biogerontological research therefore is how "healthy" aging differs from the aging seen in various neurodegenerative diseases. Distinguishing aging-associated pathological processes from beneficial ones could be important for both the further development of therapeutic treatments to neurodegenerative disease, as well as for better maintaining brain health in normal aging.

Evidence now indicates that neurons themselves, or disruptions in neuronal communication, are involved in several pathological processes that resemble those observed during aging. One example is the balance between excitatory (glutamatergic) and inhibitory (GABAergic) neurotransmission, often referred to as the E/I balance. Several rodent models have shown a correlation between decline of the glutamatergic system and age-associated decline in cognitive functions [3]. Altered glutamatergic neurotransmission can occur due to brain morphological changes that become more prominent with age, such as cortical thinning and synaptic pruning [3]. These changes in glutamate levels occur in the hippocampus, prefrontal cortex, and motor and sensory areas [4]. Kaiser et al. [5] have shown that, in healthy individuals, the glutamate concentration in the motor cortex is lower in elderly individuals compared to younger ones. This decrease seems to correlate with the reduction in brain volume [6]. Furthermore, there is substantial evidence demonstrating dysregulation of the glutamatergic system in AD, leading to excessive neuronal excitation [3]. It is likely that AD-associated proteins, such as amyloid beta and tau, contribute to this dysregulation. Moreover, glutamatergic activity decreases later in AD [3], resembling the age-related decline. However, it should be noted that most of these studies have had relatively small sample sizes, and therefore, data from larger populations of older individuals would be required to fully understand the significance of the E/I balance in the aging process in humans.

Neurons do not operate in isolation, but rather in concert with glial as well as vascular cells, and thus these other cell types should also be considered. The disruption of the delicate balance between these cell types is associated with adverse outcomes such as loss of blood-brain barrier (BBB) integrity and neuroinflammation, and can lead to dendritic loss and synaptic nerve damage [7]. Glial cells include such cell types as oligodendrocyte lineage cells, astrocytes and microglia. Glial cells provide nutrition and tropic support to neurons, but they may have more direct involvement with them as well. For example, in the concept of “tripartite synapses”, astrocytes are included in the synaptic model, regulating neuronal activity in a feedback manner after receiving neuronal signals [8]. Recent evidence from multiple studies shows that microglia may also be involved in regulation of synaptic function by the secretion of various substances, such as tumor necrosis factor alpha (TNF-α), interleukin-1β (IL-1β), and brain-derived neurotrophic factor (BDNF) [8]. Senescence of glial cells may be connected to inflammaging, by causing a more inflammatory microenvironment and leading to neuron and synapse loss [9]. Vascular cells such as endothelial cells are also necessary for neuronal and glial cell function. Endothelial cells are critical components of the BBB that regulates the exchange of substances between the blood and the brain, and BBB dysfunction is a major player in AD pathology [10].

There is evidence that sex differences also play a significant role in neuroinflammation and neurodegeneration. The sexes have been found to have differences in the brain that are both structural and neurochemical, including differences in GABAergic markers [11]. In AD, age and sex both are thought to drive hallmark β-amyloid (Aβ) neuropathology [12], involving the accumulation and deposition of β-amyloid protein plaques in the brain. One suggested explanation for this association relates to Aβ aggregation by Zn^2+^ released from glutamatergic neurons [13]. The higher risk of AD could thus be caused by age-associated impairment of Zn^2+^ reuptake and the higher maximal capacity for Zn^2+^ in females [13]. If the mechanisms underlying these sex differences could be determined, it would benefit the development of targeted therapeutic strategies that consider the sex-specific aspects of neurodegenerative diseases.

In this study we have analyzed the effects of age and sex differences on brain cell composition in order to investigate the potential roles of the studied cell types in aging and in aging-associated pathology. As materials we utilized a large dataset (n = 1646) of RNA sequenced brain samples from the Genotype-Tissue Expression (GTEx) project [14]. Our methods centered around RNA sequencing (RNA-seq) based cell type deconvolution with the digital cytometry tool CIBERSORTx [15]. In this approach, gene expression data is compared to previously established gene expression profiles of different cell types to estimate the proportions of cell types present in each sample.

## Materials and methods

### Data

Genotype-Tissue Expression project (GTEx) raw gene expression read count data was used (dbGaP Accession phs000424.v8.p2) [14]. In the GTEx project, RNA-seq was performed using the Illumina TruSeq library construction protocol (non-stranded, polyA+ selection). Alignment to the human reference genome GRCh38/hg38 was performed using STAR v2.5.3a, based on the GENCODE v26 annotation. The full GTEx alignment pipeline is available at https://github.com/broadinstitute/gtex-pipeline/tree/master/rnaseq (URL last accessed on 2nd October 2024). As part of expression quantification in the GTEx project, only reads meeting the following criteria were kept:

1. Reads were uniquely mapped (corresponding to a mapping quality of 255 for STAR BAMs).
2. Reads were aligned in proper pairs.
3. The read alignment distance was < = 6 (i.e., alignments must not contain more than six non-reference bases).
4. Reads were fully contained within exon boundaries. Reads overlapping introns were not counted.

More detailed information on the data generation and processing in the GTEx project can be found in the GTEx portal at https://gtexportal.org/home/methods (URL last accessed on 2nd October 2024).

We utilized the RNA sequencing data from all brain samples from GTEx that were aligned to the Genome Reference Consortium Human Build 37 (*GRCh37*) and are not technical controls. This resulted in RNA-seq data from a total of 1646 samples. The samples originate from 13 different brain regions. The number of samples per region are as follows: cerebellum 167, cortex 156, caudate (basal ganglia) 154, nucleus accumbens (basal ganglia) 148, cerebellar hemisphere 134, frontal cortex (BA9) 131, putamen (basal ganglia) 124, hypothalamus 123, hippocampus 120, anterior cingulate cortex (BA24) 115, amygdala 100, spinal cord (cervical c-1) 89, substantia nigra 85. The samples are derived from a total of 249 individuals, with up to 13 samples from the same individual in some cases. Each sample donor provided at most one sample for each brain region. The sample donors are between 20 and 70 years of age. Samples originate from both female and male donors, with 493 samples from female and 1153 from male donors. All GTEx sample donors were surgical patients or post-mortem donors, and the samples are derived from non-diseased tissue.

We obtained the raw RNA-seq gene level read counts for the 1646 brain samples from the GTEx Portal on 7^th^ of March 2023. These raw read counts had not been normalized or corrected for any covariates. We CPM (Counts Per Million) normalized the read counts to match the normalization of the signature matrix used in cell proportion deconvolution and to adjust for differences in sequencing depth across samples. Furthermore, when deconvolution of the CPM normalized read counts was performed utilizing the tool CIBERSORTx, batch correction was enabled to minimize technical biases and ensure that cell-type proportion estimates are as accurate as possible.

### Age group subsets

For the benefit of certain analyses, the samples were divided into two age groups: the young and the old. Donors between the ages of 20 and 35 years were considered young, while donors between 60 and 70 years of age were considered old. There was a total of 94 samples from young donors and 840 samples from old donors. Of the young donor originating samples, 63 are from male donors and 31 from female donors. Of the old donor originating samples, 576 are from male donors and 264 from female donors.

### Signature matrix origin

The signature matrix originates from a 2020 study by Wang et al [16]. The signature matrix was built by Wang et al. utilizing adult scRNA-seq data from Darmanis et al. [17], as well as marker genes from the PsychENCODE Consortium [18] and microglia markers from Olah et al. [19]. More detailed information on the signature matrix creation can be found in the original Wang et al. article [16].

### Brain cell type deconvolution

The proportions of brain cells were estimated using deconvolution of RNA-seq data based on the gene expression of marker genes with the software tool CIBERSORTx [15]. The raw gene expression read counts were first obtained from the GTEx datasets. The counts were then CPM (Counts Per Million) normalized and log2 transformed to match the data processing of the signature matrix by Wang et al. [16]. Normalization was done using the R programming language. CIBERSORTx was run utilizing this normalized data as the mixture matrix to determine the proportions of brain cell types for each sample. Cell type proportions were estimated for excitatory and inhibitory neurons, astrocytes, oligodendrocytes, microglia, and endothelial cells. CIBERSORTx batch correction was enabled. The number of permutations was set to 1000 for CIBERSORTx deconvolution significance analysis to verify the success of the deconvolution of each sample.

### Correlation analysis

Associations between variables were studied using Spearman rank correlation coefficient. Analysis was performed using the R programming language.

### Evaluation of the distribution of donor and sample characteristics

Further statistical testing was conducted to verify that there are no sampling biases between ages, sexes or originating brain regions that could confound the results. To test whether there could be a significant difference in ages of sample donors between males and females, a Mann-Whitney U-test was used. Mann-Whitney U-test was utilized due to the ages of both males and females having a non-Gaussian distribution as indicated by Shapiro-Wilk tests of normality (p-values < 0.05). As the number of samples originating from male donors (n = 1153) is higher than that from female donors (n = 493), bootstrap resampling without replacement was performed to investigate whether the observed sex differences could be due to sample size difference. This was implemented by comparing the results from the full male dataset to the median results of 1000 iterations of randomly sampled sets of male samples equal in size to the number of female originating samples (n = 493). Additionally, a Chi-Squared test was used to determine whether there is a significant difference (p-value threshold 0.05) between the sexes in the numbers of samples from different brain regions. All these analyses were performed using the R programming language.

### R programming language and RStudio versions

All R scripting was done utilizing the R version 4.3.1 (2023-06-16 ucrt) and RStudio version 2024.04.2 Build 764. Code utilized in the analyses can be found in S1 File.

## Results

**Fig 1:**
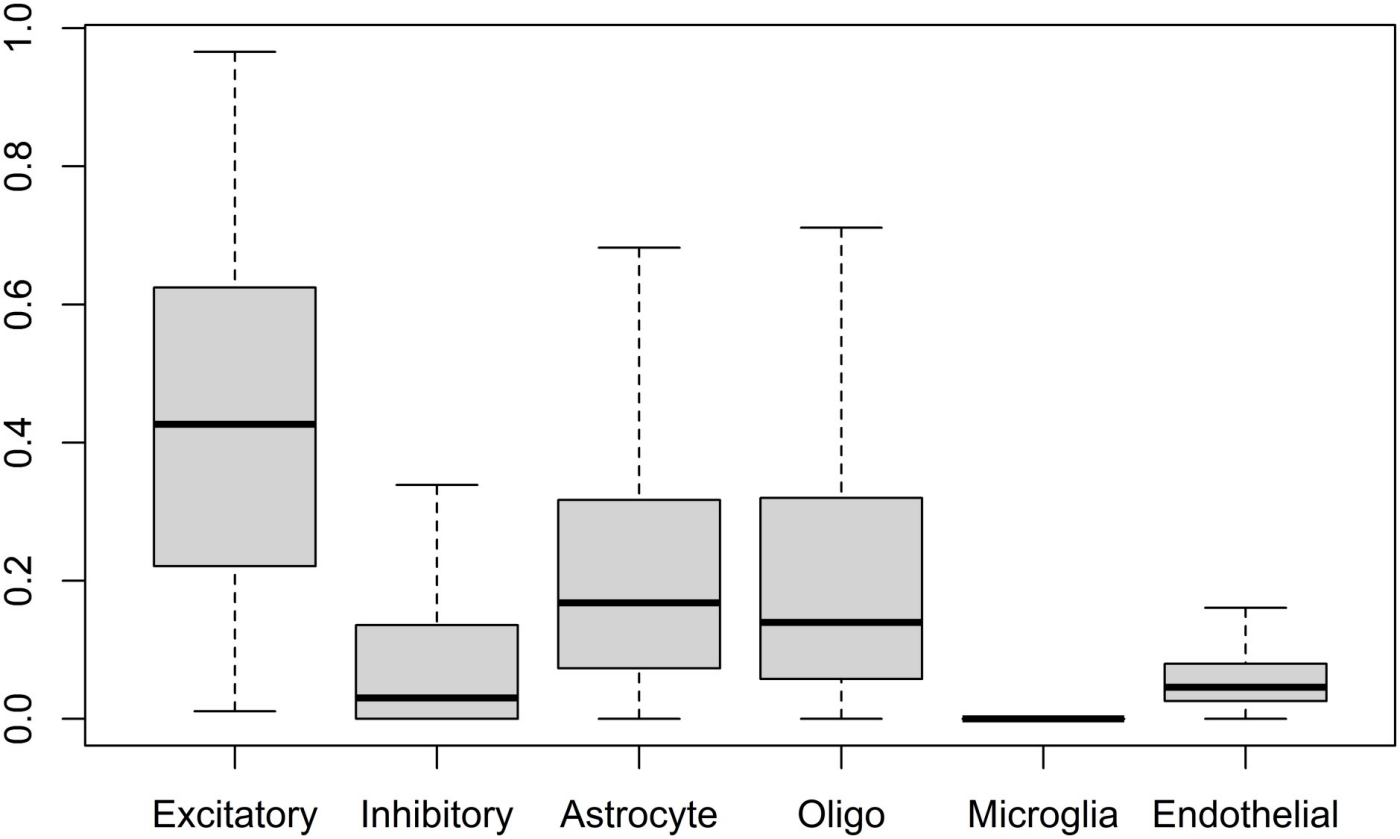
Box plot displaying the distributions of the deconvolution derived proportions of cell types. Quantitated cell types were neuronal (excitatory, inhibitory), glial (astrocyte, oligodendrocyte, microglia), and endothelial cells. Utilized dataset consisted of 1646 brain samples of the studied GTEx data. In the box plot the median is represented by a horizontal line inside the box, while the boxes represent the interquartile range spanning the central 50% of the dataset, and the whiskers extend from the box and indicate the range of the data. Outliers are omitted from the figure to improve clarity.

Deconvolution based analysis of neuronal, glial and endothelial cell type proportions was performed on the RNA-seq data from 1646 samples of the studied GTEx data. A box plot summarizing the results is shown in Fig 1. The results indicate that in the studied samples the average cell proportions were as follows from most to least numerous: excitatory neurons (42.7%), astrocytes (21.0%), oligodendrocytes (22.1%), inhibitory neurons (8.0%), endothelial cells (6.1%), and microglia (0.1%).

**Fig 2:**
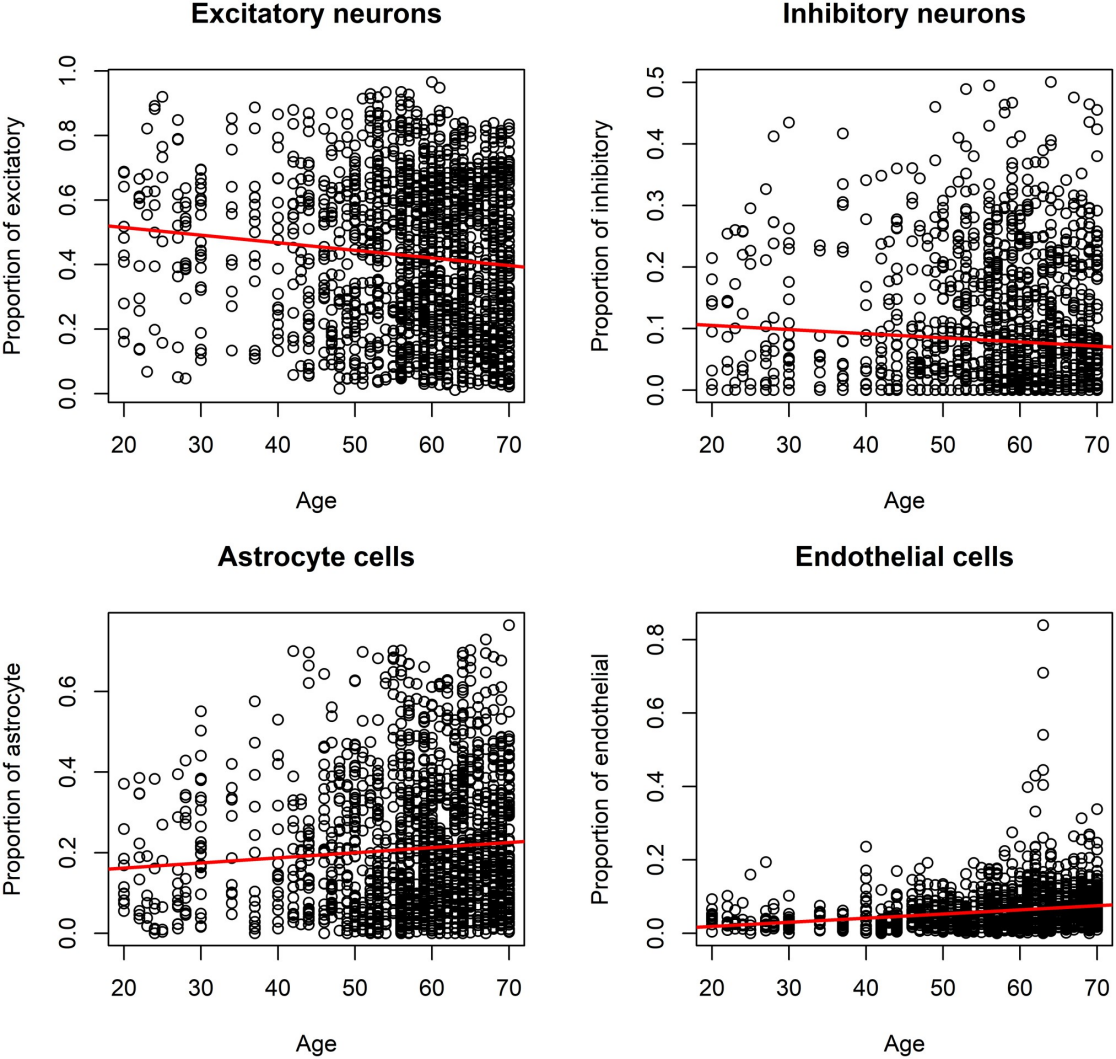
Scatter plots of the relationships between age and the proportions of cells which statistically significantly correlated with age. P-values smaller than 0.05 were considered significant, and significant results included neurons (both excitatory and inhibitory), astrocytes, and endothelial cells. The proportions are based on the share of the individual cell type from the studied 6 cell types.

As age is a prominent risk factor in various neuroinflammatory diseases, the potential association between sample donor age and the estimated cell type proportions was investigated. Spearman correlation test was performed on calendar ages of the individuals compared to the abundances of the quantified cell type proportions. The results, as shown in Table 1 and Fig 2, show that the proportion of neurons statistically significantly decreased with age, while the proportions of astrocytes and endothelial cells significantly increased (p-values < 0.05). The strongest correlation coefficient (rho = 0.29) was seen with endothelial cells, which had a positive correlation with age.

**Table 1:** Spearman correlations between the age of the sample donor (20–70 years) and estimated proportions of neuronal, glial, and endothelial cells. Statistically significant p-values (p-value < 0.05) are marked with *. Also shown are the median and mean proportions, showing proportionate prevalence (between 0 and 1) of each cell type out of all cells. These statistics are shown both for all sample donors and separately for male and female sample donors.

Cell type	All sample donors				Male				Female			
	rho	p-value	mdn	mean	rho	p-value	mdn	mean	rho	p-value	mdn	mean
Excitatory neurons	-0.11	< 0.001*	0.43	0.43	-0.13	< 0.001*	0.44	0.44	-0.06	0.157	0.40	0.41
Inhibitory neurons	-0.09	< 0.001*	0.03	0.08	-0.10	< 0.001*	0.03	0.08	-0.08	0.096	0.02	0.08
Total neurons	-0.13	< 0.001*	0.52	0.51	-0.15	< 0.001*	0.54	0.52	-0.09	0.052	0.50	0.48
Astrocytes	0.09	< 0.001*	0.17	0.21	0.12	< 0.001*	0.17	0.21	0.04	0.408	0.17	0.22
Oligodendrocytes	0.03	0.188	0.14	0.22	0.04	0.143	0.14	0.22	0.01	0.795	0.14	0.23
Microglia	0.03	0.187	0.00	0.00	-0.01	0.769	0.00	0.00	0.11	0.013*	0.00	0.00
Endothelial cells	0.29	< 0.001*	0.05	0.06	0.26	< 0.001*	0.04	0.06	0.35	< 0.001*	0.05	0.07

As cell type proportions are connected to the sex of the individual, we also analyzed males and females separately in terms of aging-associated effects. The male and female sample donor age distribution is comparable, as Mann-Whitney U-test indicates that there is no significant difference in age between the sexes (p-value = 0.370). There is also no significant difference between the sexes in the originating brain regions of the samples, as indicated by a Chi-Squared test (p-value = 0.924).

Analyzing the sexes separately for associations between cell type proportions and age, we found that the age-associated statistically significant decrease in neurons was only seen in male donors, not in female donors (Table 1). In addition, a significant age-associated increase in astrocyte proportions was only seen in males, while only females had a significant increase in microglia. These results are robust and persist when accounting for the sample size difference between males (n = 1153) and females (n = 493). This was tested with bootstrap resampling of n = 493 sized male datasets, equal in number of samples to the female dataset. The bootstrapped median results of 1000 iterations indicated statistically significant associations between age and the same cell types as the full male dataset, shown in detail in Table 2.

**Table 2:** Bootstrapped spearman correlations from a randomly selected subset of male sample donor originating samples (n = 493). Correlations are analyzed between the age of the sample donor (20–70 years) and estimated proportions of neuronal, glial, and endothelial cells. Statistically significant p-values (p-value < 0.05) are marked with *. Additionally included are the median and mean proportions, showing proportionate prevalence (between 0 and 1) of each cell type out of all cells.

Cell type	Male			
	Rho	P-value	Mdn	Mean
Excitatory neurons	-0.13	0.004*	0.45	0.43
Inhibitory neurons	-0.10	0.023*	0.04	0.09
Total neurons	-0.15	0.001*	0.56	0.52
Astrocytes	0.12	0.009*	0.17	0.21
Oligodendrocytes	0.05	0.309	0.13	0.23
Microglia	-0.01	0.544	0.00	0.00
Endothelial cells	0.26	< 0.001*	0.04	0.06

An age-associated difference in E/I (excitatory neuron / inhibitory neuron) balance was also observed. For men, there was a statistically significant, though weak, positive correlation between age and E/I value (p-value = 0.013, rho = 0.07). For women, a similar effect was seen, though it was not statistically significant (p-value > 0.05, rho = 0.07). The lack of statistical significance in the case of women could be due to smaller sample size. This was tested with bootstrap resampling of n = 493 sized male datasets, equal in number of samples to the female dataset. The bootstrapped median results of 1000 iterations of downsampled male datasets indicate no statistically significant association between age and E/I value (p-value > 0.05, rho = 0.07).

**Fig 3:**
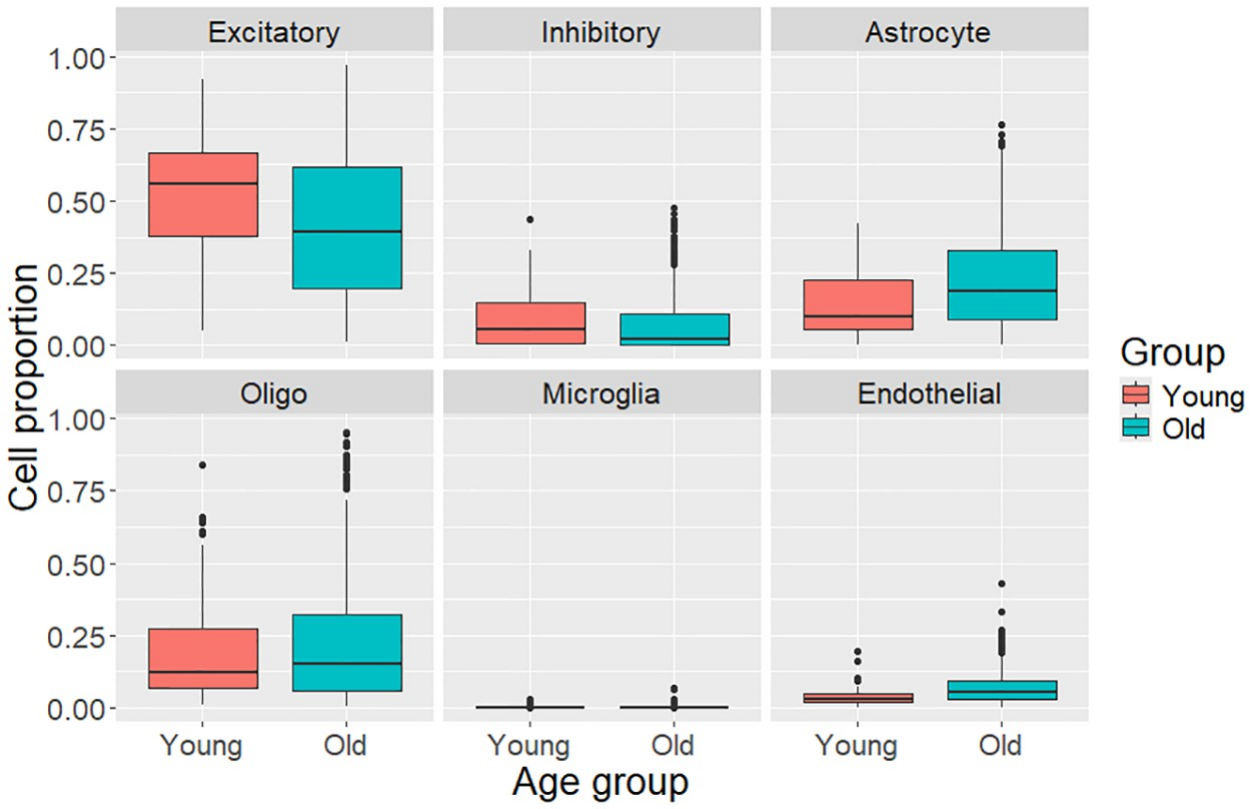
Box plots for subsets of male originating samples, displaying the distributions of the proportions of neuronal, glial, and endothelial cells. Young are 20–35 years of age and old are 60–70 years of age. All cell types had significant age-association (p-value < 0.05), except for oligodendrocytes.

**Fig 4:**
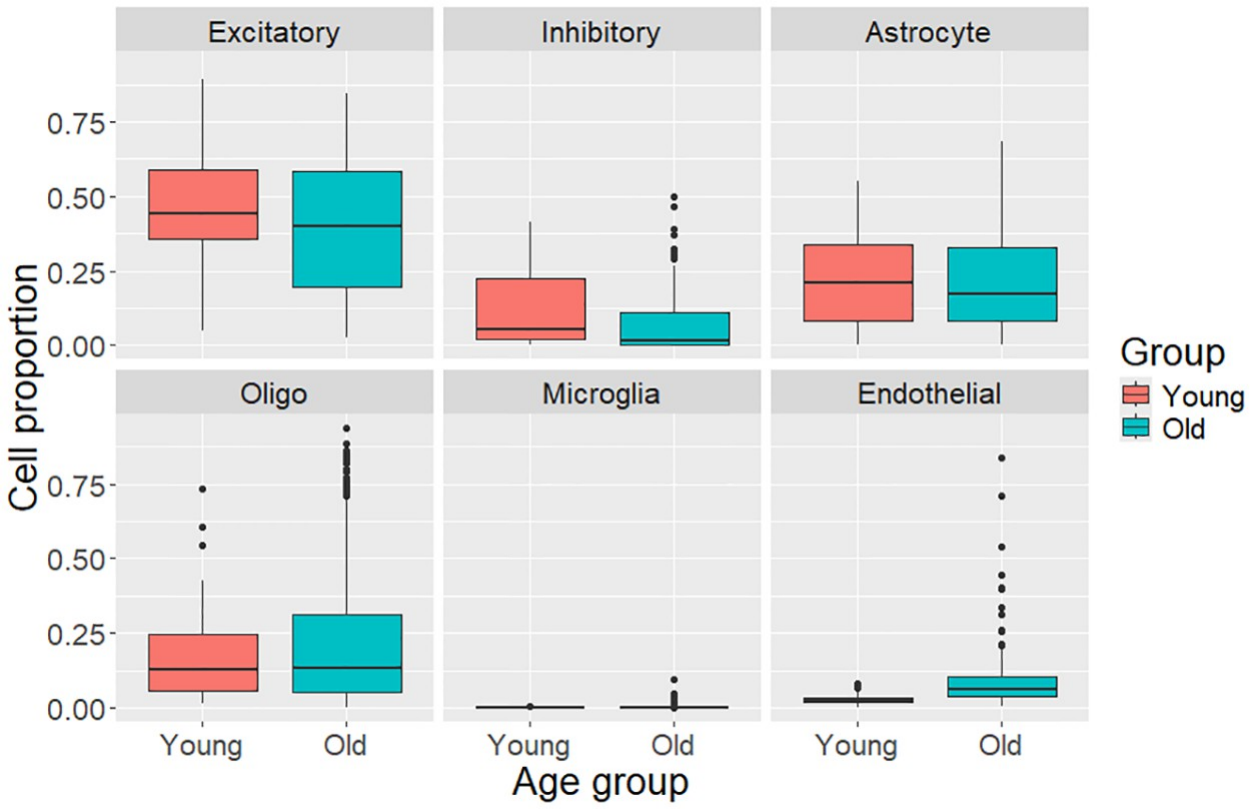
Box plots for subsets of female originating samples, displaying the distributions of the proportions of neuronal, glial, and endothelial cells. Young are 20–35 years of age and old are 60–70 years of age. Significant age-associated differences were seen in the proportions of inhibitory, microglia, and endothelial cells.

In addition to the correlation-based study of aging-associated effects, analyzes were also done on two subsets of samples from young and old age groups. Young was considered to be 20–35 years of age and old to be 60–70 years of age. For men, all cell types except for oligodendrocytes had significant age-associated changes (p-value < 0.05). For women, significant age-associated differences were seen in the proportions of inhibitory, microglia, and endothelial cells. Results are displayed in Figs 3 and 4.

These sex-based differences in cell type proportions also extend outside the aging process. Statistically significant differences between male and female sample donors were seen using Mann-Whitney U-test in estimated proportions of excitatory neurons (p-value = 0.018), total neurons (p-value = 0.014), microglia (p-value = 0.016), and endothelial cells (p-value < 0.001). Male samples had higher proportions of excitatory neurons and female samples had higher proportions of microglia and endothelial cells.

As the data is derived from samples originating from 13 different brain regions, region specific analyses of the effect of age and sex were also performed. For men, region-specific correlations between age and proportion of brain cell types are shown in Table 3. For women, significant region-specific correlation was only seen in endothelial cells and in samples originating in the cortex (p-value = 0.018, rho = 0.51). Additionally, region-specific differences between the sexes outside of age-association were also observed, with results shown in Table 4. Region-specific significant differences were seen in proportions of excitatory neurons, total neurons, microglia and endothelial cells.

**Table 3:** Spearman correlation of age with proportions of cell types in men, for each brain region. Shown p-values are Benjamini-Hochberg corrected. Only significant results (p-value < 0.05) are shown.

Tissue	Cell type	P-value	Rho
Amygdala	Oligo	0.025	0.35
Amygdala	Endothelial	0.038	0.33
Anterior cingulate cortex (BA24)	Endothelial	0.025	0.32
Caudate (basal ganglia)	Endothelial	0.025	0.28
Cerebellar Hemisphere	Inhibitory	0.046	-0.26
Cerebellar Hemisphere	Oligo	0.025	-0.31
Cerebellar Hemisphere	Endothelial	0.025	0.29
Cerebellum	Endothelial	0.025	0.29
Cortex	Excitatory	0.038	-0.26
Cortex	Neurons_total	0.041	-0.25
Frontal Cortex (BA9)	Excitatory	0.041	-0.27
Frontal Cortex (BA9)	Inhibitory	0.040	-0.28
Frontal Cortex (BA9)	Neurons_total	0.025	-0.31
Frontal Cortex (BA9)	Oligo	0.049	0.26
Frontal Cortex (BA9)	Endothelial	0.041	0.27
Hippocampus	Excitatory	0.012	-0.38
Hippocampus	Neurons_total	0.012	-0.39
Hippocampus	Endothelial	0.000	0.51
Hypothalamus	Astrocyte	0.025	0.32
Hypothalamus	Endothelial	0.025	0.34
Nucleus accumbens (basal ganglia)	Endothelial	0.025	0.30
Putamen (basal ganglia)	Endothelial	0.038	0.29
Spinal cord (cervical c-1)	Inhibitory	0.049	-0.34

**Table 4:** Sex-based differences for combinations of brain regions and cell types, based on Mann-Whitney U-test. Shown p-values are Benjamini-Hochberg corrected. Only significant results (p-value < 0.05) are shown.

Tissue	Cell type	P-value	Male mdn	Female mdn
Amygdala	Excitatory	0.031	0.34	0.37
Amygdala	Neurons_total	0.031	0.37	0.38
Amygdala	Microglia	0.031	0.00	0.00
Amygdala	Endothelial	0.006	0.06	0.05
Anterior cingulate cortex (BA24)	Excitatory	0.031	0.58	0.53
Anterior cingulate cortex (BA24)	Neurons_total	0.031	0.60	0.55
Anterior cingulate cortex (BA24)	Microglia	0.031	0.00	0.00
Anterior cingulate cortex (BA24)	Endothelial	0.006	0.04	0.04
Caudate (basal ganglia)	Excitatory	0.031	0.25	0.27
Caudate (basal ganglia)	Neurons_total	0.031	0.32	0.32
Caudate (basal ganglia)	Microglia	0.031	0.00	0.00
Caudate (basal ganglia)	Endothelial	0.006	0.06	0.06
Cerebellar Hemisphere	Excitatory	0.031	0.64	0.61
Cerebellar Hemisphere	Neurons_total	0.031	0.90	0.87
Cerebellar Hemisphere	Microglia	0.031	0.00	0.00
Cerebellar Hemisphere	Endothelial	0.006	0.03	0.02
Cerebellum	Excitatory	0.031	0.62	0.60
Cerebellum	Neurons_total	0.031	0.81	0.77
Cerebellum	Microglia	0.031	0.00	0.00
Cerebellum	Endothelial	0.006	0.04	0.05
Cortex	Excitatory	0.031	0.68	0.68
Cortex	Neurons_total	0.031	0.69	0.68
Cortex	Microglia	0.031	0.00	0.00
Cortex	Endothelial	0.006	0.03	0.04
Frontal Cortex (BA9)	Excitatory	0.031	0.72	0.72
Frontal Cortex (BA9)	Neurons_total	0.031	0.74	0.73
Frontal Cortex (BA9)	Microglia	0.031	0.00	0.00
Frontal Cortex (BA9)	Endothelial	0.006	0.02	0.03
Hippocampus	Excitatory	0.031	0.41	0.31
Hippocampus	Neurons_total	0.031	0.42	0.32
Hippocampus	Microglia	0.031	0.00	0.00
Hippocampus	Endothelial	0.006	0.06	0.07
Hypothalamus	Excitatory	0.031	0.25	0.23
Hypothalamus	Neurons_total	0.031	0.54	0.49
Hypothalamus	Microglia	0.031	0.00	0.00
Hypothalamus	Endothelial	0.006	0.07	0.08
Nucleus accumbens (basal ganglia)	Excitatory	0.031	0.41	0.36
Nucleus accumbens (basal ganglia)	Neurons_total	0.031	0.50	0.45
Nucleus accumbens (basal ganglia)	Microglia	0.031	0.00	0.00
Nucleus accumbens (basal ganglia)	Endothelial	0.006	0.03	0.04
Putamen (basal ganglia)	Excitatory	0.031	0.22	0.21
Putamen (basal ganglia)	Neurons_total	0.031	0.26	0.24
Putamen (basal ganglia)	Microglia	0.031	0.00	0.00
Putamen (basal ganglia)	Endothelial	0.006	0.06	0.06
Spinal cord (cervical c-1)	Excitatory	0.031	0.05	0.06
Spinal cord (cervical c-1)	Neurons_total	0.031	0.05	0.06
Spinal cord (cervical c-1)	Microglia	0.031	0.00	0.00
Spinal cord (cervical c-1)	Endothelial	0.006	0.07	0.08
Substantia nigra	Excitatory	0.031	0.15	0.19
Substantia nigra	Neurons_total	0.031	0.20	0.24
Substantia nigra	Microglia	0.031	0.00	0.00
Substantia nigra	Endothelial	0.006	0.09	0.10

## Discussion

The results indicate notable age-associated changes to brain cell composition, including a statistically significant decrease in both excitatory and inhibitory neurons. Our results are in line with a previous report by Kaiser et al. [5], which relates to the phenomenon of age-associated neuronal loss. They used magnetic resonance spectroscopy to analyze the effect of aging on glutamate concentration in the brain of healthy individuals (age range 24–68 years of age) and observed that older subjects had lower levels. Glucose metabolism is critical for neurotransmitter synthesis and homeostasis, especially in the glutamatergic and GABAergic systems.

The strongest association between age and a cell type was seen with the positive correlation between age and proportions of endothelial cells in the brain, which could have significant implications. An increase in the proportion of endothelial cells with age could affect the balance of neural cells. It could also indicate an age-associated decline in endothelial cell function, leading to an increase in the number of endothelial cells to compensate for the decline in function. Brain endothelial cells are an important part of the blood-brain barrier [20]. Therefore, the dysfunction of brain endothelial cells could compromise the function of the BBB. This could allow more harmful substances through, such as pathogens and inflammatory cells. Moreover, many therapeutics struggle to cross the intact BBB, and if the barrier becomes compromised with age or disease, it may further impede the delivery of drugs to the brain, limiting their effectiveness in treating brain disorders. Damage to the BBB is indeed a common feature in many neuroinflammatory conditions [20], and neurodegenerative diseases are overall associated with alterations in vascular function and cerebral blood flow [21].

The results also indicate a statistically significant increase in astrocytes with age. Considering that astrocytes are closely involved in neuron function and the BBB, alterations to their function could have impactful consequences. Furthermore, astrocytes have been found to become more pro-inflammatory with age and to contribute to brain inflammaging [22].

Analysis of the cell type proportions from male and female sample donors separately suggest a sex-based difference in the relationship between age and neural cell proportions. The age-associated statistically significant decrease in neurons was only observed in male donors. A review article [22] has noted that males have been reported to have a greater loss of brain volume with age than females [23] and that this decrease in volume has been hypothesized to be due to neuron loss. Additionally, the age-associated increase in microglia proportion that was only observed in females could be worth further investigation considering the both neuroprotective and neuroinflammatory potential of microglia [8] and the higher risk of Alzheimer’s for females [13]. The results from the analysis of two subsets of samples, those from young (here defined as 20–35 years of age) and old (60–70 years of age), showed similar results to the correlation-based analysis.

A significant age-associated difference in E/I balance was seen in male sample donors, but not in female sample donors. However, our data does not support the conclusion that this is necessarily a true difference between the sexes, according to a downsampling experiment. The bootstrap resampling of male datasets, equal in number of samples to the female dataset, indicate no significant association between age and E/I ratio in males. Thus, the initial difference in results between the sexes can be explained by the larger number of samples from male donors. It would be interesting to see if a sufficiently large dataset from female sample donors would show a significant age-associated difference in E/I balance, similarly to the full male dataset.

The observed differences between female and male sample donors indicate that the cell type proportions vary between sexes, not only during the aging process, but also more broadly. Male samples had higher proportions of excitatory neurons and female samples had higher proportions of microglia and endothelial cells. Differences have been found in microglia and neuroimmune signaling between male and female brains, and microglia have even been suggested to have a central role in sex differences relating to incidence of neuropsychiatric and neurological disorders [24].

Similarly to the region combined dataset, brain region specific differences were also seen in proportions of excitatory, total neuron, microglia and endothelial cells. Notably the direction of the difference varies by brain region, with males having higher proportions of certain cells in certain regions and vice-versa. This indicates that age-associated changes in cell composition may be brain region specific and that specific brain regions of males and females could age differently.

There was a large amount of variation in estimated cell proportions of different cell types between samples. The variation was greatest in the proportion of excitatory cells between samples, as can be seen in Figs 1, 3 and 4. This high variation in excitatory cells could be due to the data originating from different brain regions or even areas within these regions, as abundance of excitatory neurons is known to vary between different areas of the brain. A large amount of variance is common to deconvolution results, as can be seen in Fig 2b in a deconvolution algorithm comparison study by Patrick et al. [25].

One limitation of this study is the relative nature of the cell type proportion estimations, as they are proportions instead of absolute values. Obtaining absolute gene expression values directly from RNA-seq data is challenging due to various technical factors involved in the sequencing process and data analysis. The cell type deconvolution results analyzed here are based on gene expression values from RNA-seq that indicate relative rather than absolute gene expression. The resulting relative proportions of cell types limits comparison between age-associated changes in cell types, as an increase in one cell type proportion will always be accompanied by a decrease in other cell type proportions. Future analysis of absolute cell type proportions could yield valuable additional information of associations between cell types.

Though beyond the scope of this work, use of more specific reference datasets in future studies could improve the accuracy of the deconvolution results. Reference datasets, in the form of signature matrices in the CIBERSORTx deconvolution software tool, are limited in availability. CIBERSORTx is intended to be robust against even the presence of unknown cell types in the samples [15], yet the use of more specific datasets could yield more precise and accurate results. Use of age-specific and sex-specific reference datasets for deconvolution could mitigate potential bias from age- and sex-associated expression differences in specific cell types. Similarly, the age range of sample donors in the reference dataset could be considered, to match with the age range of studied samples. Furthermore, cell type compositions vary between brain regions, and therefore even brain region specific reference datasets could allow more accurate deconvolution results. The potential issues and limits of reference datasets have been previously discussed by Patrick et al. [25].

## Conclusions

Our results show that both age and sex affect the proportions of neurons, glial cells and endothelial cells in non-diseased brain tissue samples. Our brain-wide analyses indicate that there is an age-associated increase in the proportions of astrocytes and endothelial cells, and an age-associated decrease in neurons, both excitatory and inhibitory. Between men and women, statistically significant differences were seen in the proportions of the studied brain cells. Women had a higher number of endothelial and microglia cells on average, while men had a higher number of excitatory cells and total neuron cells. When the aging-associated changes were studied separately for the sexes, the aging-associated changes in men are as described above, yet in women only an aging-associated increase in endothelial cells and microglia was observed. The importance of these findings relates to age and sex also having been identified as risk factors in neurological diseases. A greater understanding of such changes to brain cell proportions could help in identifying the underlying mechanisms of these diseases.

## Supporting information

S1 File Contains R code utilized in the analyses. (TXT)

## Abbreviations

AD: Alzheimer’s disease
BBB: blood-brain barrier
CPM: counts per million
RNA-seq: RNA sequencing
